# A patient with penile metastasis secondary to small cell lung cancer successfully treated with PD-1 inhibitors and chemotherapy: a case report and literature review

**DOI:** 10.3389/fonc.2025.1484365

**Published:** 2025-02-25

**Authors:** Kai-cong Zhang, Wei-jun Li, Hui-xin Xu, Hui-min Liang, Qiong Yang

**Affiliations:** ^1^ Department of Oncology, Sun Yat-Sen Memorial Hospital, Sun Yat-Sen University, Guangdong Provincial Key Laboratory of Malignant Tumor Epigenetics and Gene Regulation, Medical Research Center, Sun Yat-Sen Memorial Hospital, Sun Yat-Sen University, Guangzhou, China; ^2^ Pediatric Emergency Department, Guangzhou Women and Children’s Medical Center, Guangzhou Medical University, Guangdong Provincial Clinical Research Center for Child Health, Guangzhou, China

**Keywords:** penile metastasis, small cell lung cancer, PD-1 inhibitors, chemotherapy, case report, literature review

## Abstract

**Background:**

Penile metastasis is an uncommon condition, with most primary malignancies originating in the abdominal cavity and pelvis. There have been very few reported cases originating from lung cancer, most of squamous cell carcinoma without small cell lung cancer.

**Methods:**

We presented a case of penile metastasis secondary to small cell lung cancer, along with a review of relevant literature from the CNKI database.

**Results:**

A 73-year-old male presented with a one-month history of palpable swelling in the penis without any chest symptoms. Beside penile lesion, PET/CT imaging also revealed a lesion in the left lobe of the lung, as well as multiple enlarged lymph nodes in the left hilum, mediastinum, and left supraclavicular fossa. Fiberoptic biopsy confirmed small cell lung cancer for the pulmonary mass, while biopsies of the penile mass confirmed metastatic small cell carcinoma. The patient received first-line treatment of 6 cycles of PD-1 inhibitor (Toripalimab) combined with etoposide and cisplatin, achieving a partial response (PR). Subsequently, second-line therapy of etoposide and cisplatin regimen and later-line therapies of Irinotecan followed by Anlotinib were administered. The overall survival was approximately 2 years.

**Conclusion:**

Penile metastasis from small cell lung cancer is extremely rare. Treatment strategies based on guidelines for small cell lung cancer had been proven effective approaches.

## Introduction

Worldwide, penile tumor is a rare disease with a cumulative incidence rate of 0.09% and a mortality rate of 0.03% (1). Despite the abundant blood supply to the penis, metastasis to this organ is rare, with fewer than 600 cases reported in English literature. Limited knowledge exists regarding the characteristics, diagnosis, and treatment regimen for this disease. In this study, we presented a case of small cell lung cancer with penile nodules as the initial manifestation and provided a systematic review of penile metastases in the Chinese population, analyzing their characteristics of incidence, development, diagnosis, treatment options, and prognosis.

## Case report

**Figure 1:**
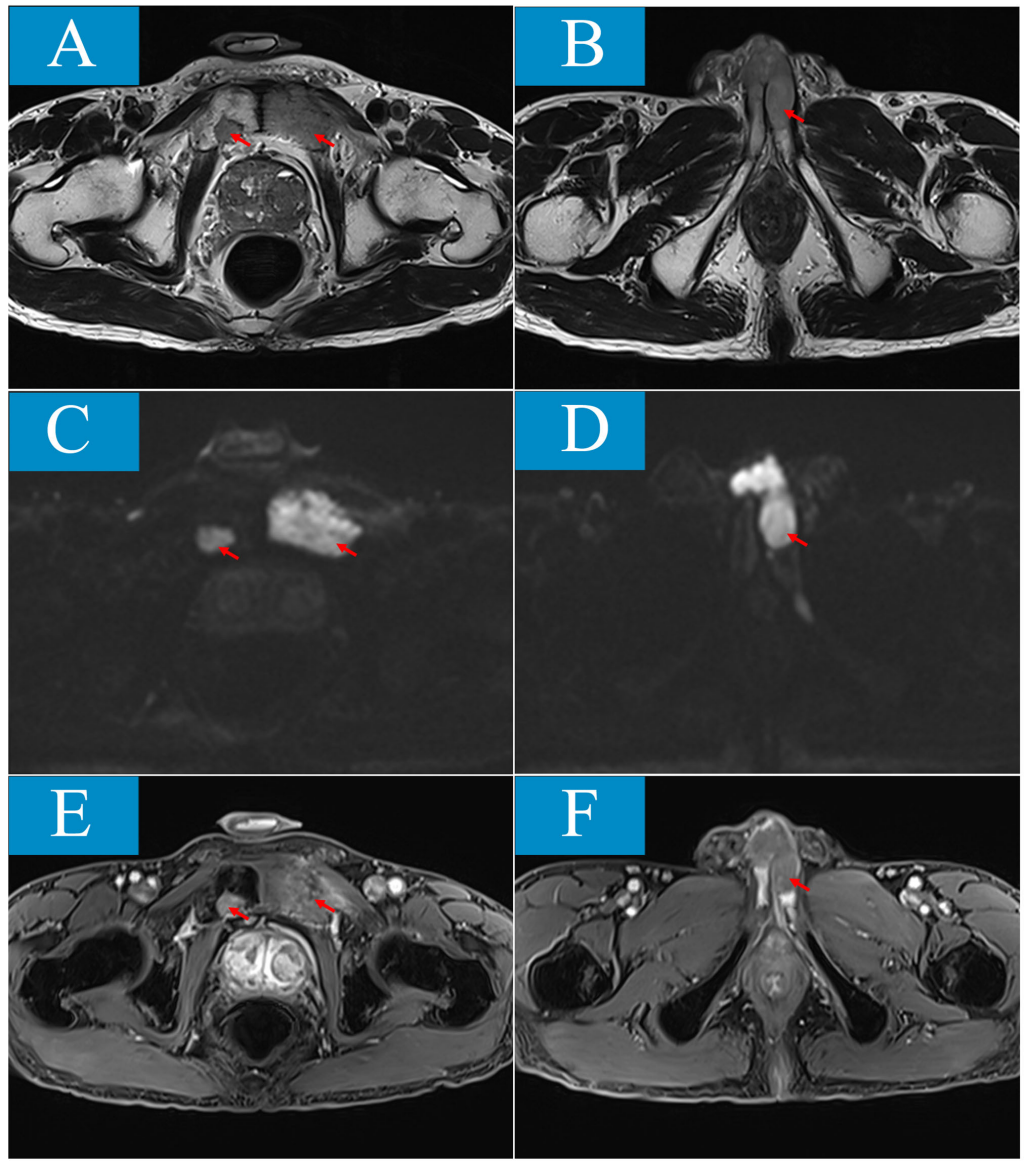
Penile and pubic lesions are showed in magnetic resonance imaging (MRI). **(A, B)** T2-weighed sequence (T2WI) shows isointense masses involving bilateral pubic body, penile corpus cavernosum and left urethracorpus cavernosum. **(C, D)** On diffusion-weighted imaging (DWI), the lesions are demonstrated limited diffuse signal intensity. **(E, F)**, After gadolinium infusion, the lesions are showed mild contrast enhancement in contrast-enhanced T1-weighted fat-suppressed sequence (T1-FS+C).

The patient, a 73-year-old heavy smoker, presented with a one-month history of palpable swelling and mild to moderate persistent pain in the perineum without malignant priapism, interrupted urine, or haematuria. No prior treatment had been administered. For the physical examination, a hard nodule measuring approximately 30*20mm was palpable at the base of the penis, while the shape of the penile shaft and glans appeared normal. There was no history of other systemic diseases. Penile ultrasound showed a greatly vascularized isoechoic mass measuring 15*14.1mm between the corpus cavernosum and urethra cavernosum. Magnetic resonance imaging (MRI) scan was performed to further characterize the lesion (**Figure 1**). The MRI findings demonstrated involvement of bilateral penile corpus cavernosum and left urethra cavernosum by multiple lesions, with the largest measuring about 32*26*58mm showing isointensity on T1 weighted imaging, slightly increased signal intensity on T2 weighted imaging, limited diffuse signal intensity on diffusion-weighted imaging (DWI), and mild contrast enhancement after gadolinium infusion. Patchy bone destruction signals were also observed in bilateral pubic bodies consistent with those seen in the penile lesion on MRI images. Additionally, an enlarged prostate measuring 57*43*50mm was detected. For tumor biomarkers, neurone specific enolase (NSE) levels were elevated at 74 ng/ml which was four times higher than its upper limit of normal (16.3 ng/ml).

**Figure 2:**
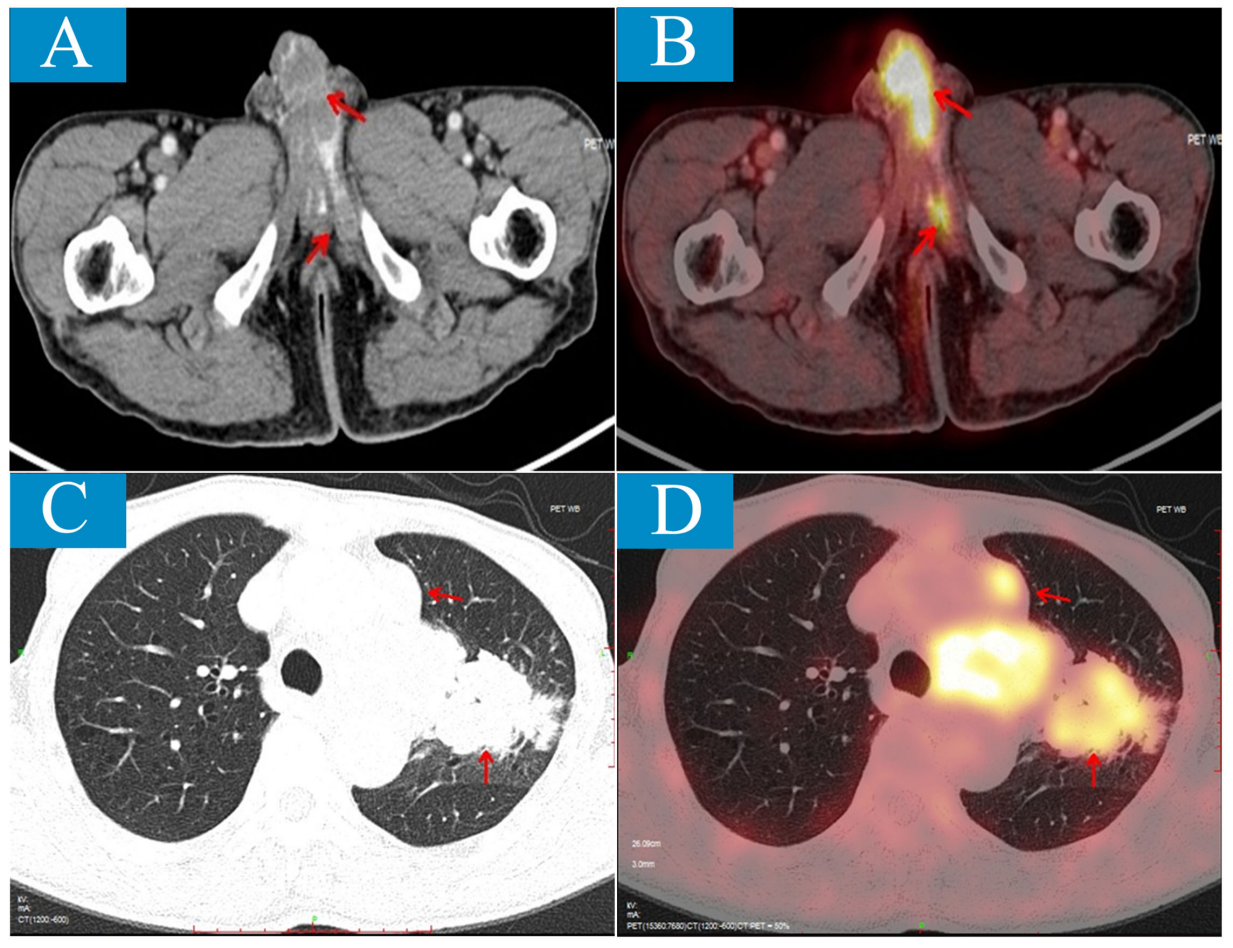
Positron emission tomography/computed tomography (PET/CT) scan shows penile metastatic lesions and primary lung lesion. **(A, B)**, Axial CT shows low-density lesions involving bilateral penile corpus cavernosum and left urethra cavernosum **(A)**; on PET/CT fusion image, the lesions show the ability of high uptake of 18F-flurodeoxyglucose (FDG) with SUVmax of 10.5 **(B)**. **(C, D)** Axial CT shows an irregular mass about 67mm×59mm×98mm located the left upper lobe near the hilum **(C)**; on PET/CT fusion image, the lesion shows the ability of high uptake of 18F-FDG with SUVmax of 9.7 **(D)**.

To investigate for other possible lesions, Positron Emission Tomography/Computed Tomography (PET/CT) scan was performed revealing not only penile and bone lesions as identified by MRI but also a left lobe lesion measuring approximately 67*59*98mm along with multiple swollen lymph nodes in the left hilum, mediastinum, and left supraclavicular fossa. The maximum standardized uptake values (SUVs) for these lesions were similar; around 10.5 for lung lesion and 9.7 for penile lesion (**Figure 2**).

**Figure 3:**
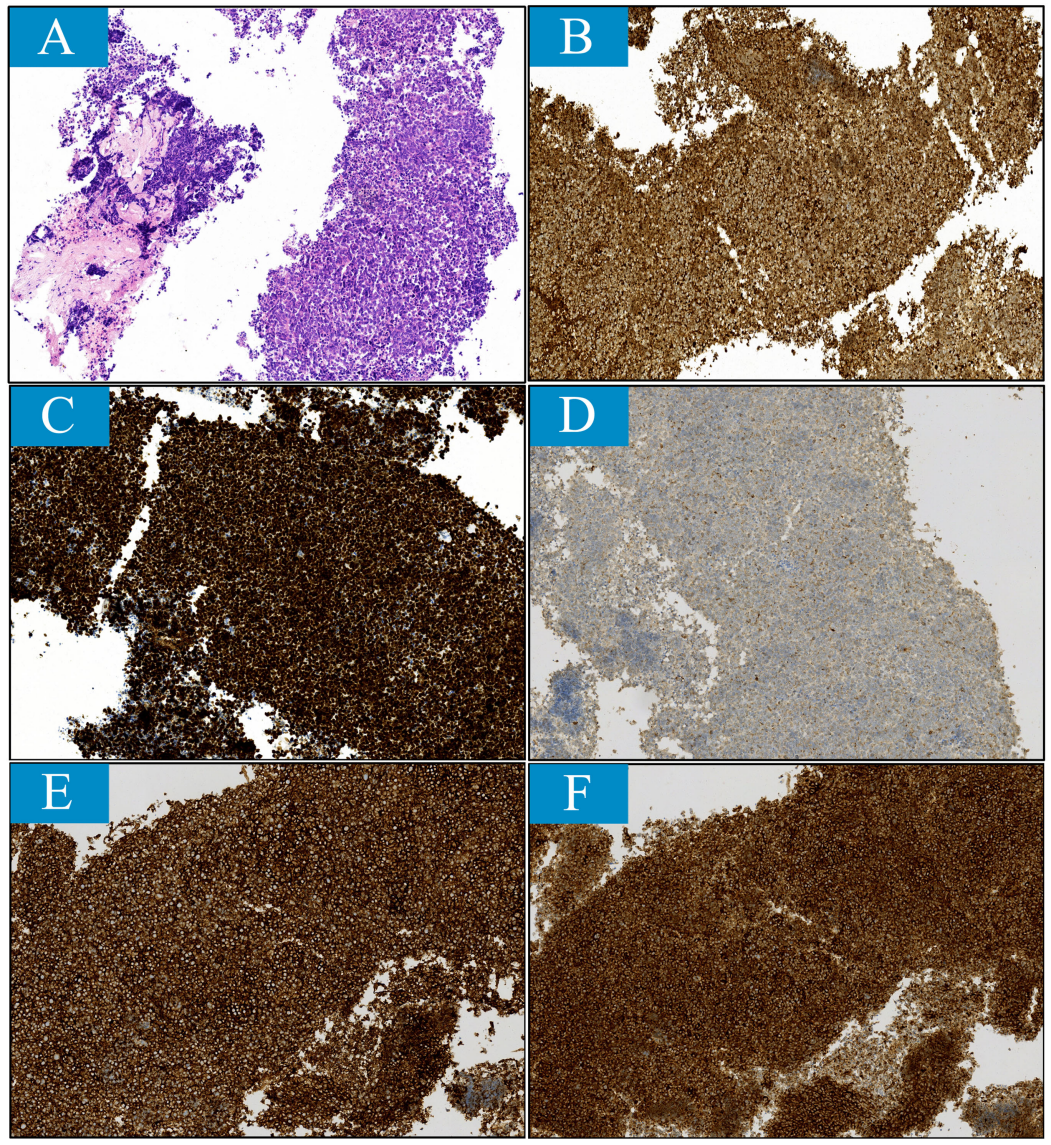
Histopathological findings reveal small cell lung cancer features for penile lesion sample obtained through fine needle aspiration (magnification ×100). **(A)** Histological feature stained with hematoxylin and eosin (H&E). **(B-F)**, Immunohistochemical features. The tumor was stained positively for **(B)** cytokerin, **(C)** thyroid transcription factor-1, **(D)** chromogranin A, **(E)** CD56 and **(F)** synaptophysin.

Given that metastasis to penis is extremely rare, pathological samples were obtained from both lung lesion via fiberoptic biopsy and penile lesion by fine needle biopsy. Histologically, the lung sample exhibited diffuse growth of malignant cells with necrosis. These cells were characterized by their small size. Immunohistochemical staining revealed positive cellular expression for cytokeratin, TTF1, NSE, Syn, CgA, and CD56 and negative for LCA and P40. In the penile sample, numerous heterotypic cells were observed with positive staining for cytokeratin, TTF1, Syn, CgA, and CD56 (**Figure 3**). These findings support the diagnosis of penile metastasis originating from small cell lung carcinoma.

**Figure 4:**
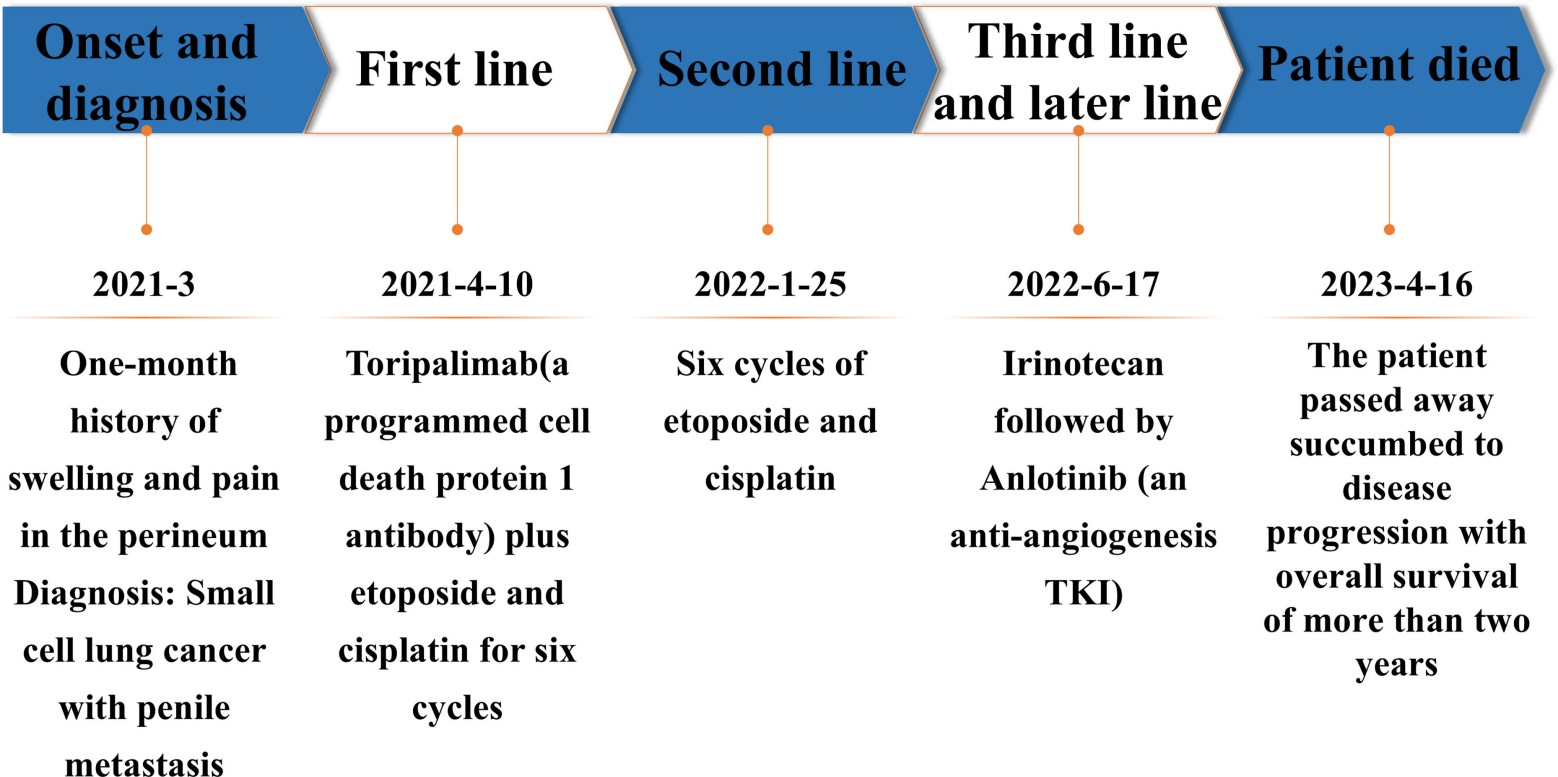
Timeline of diagnosis and treatment process.

Starting from April 10th, 2021 onwards, the patient initiated first-line anti-cancer treatment consisting of Toripalimab (a programmed cell death protein 1 antibody), along with etoposide and cisplatin. Due to existing bone metastases, zoledronic acid was administered every 28 days to prevent skeletal-related events. Pain in the perineum subsided immediately after one cycle of treatment. The patient completed a total of six cycles of treatment on August 3rd, 2021.The partial response (PR) was achieved after two cycles, and PR was confirmed after completing all six cycles. But on January 21st, 2022, a Computed Tomography (CT) scan revealed disease progression characterized by an increased number of lung metastatic lesions as well as significant enlargement of lymph nodes in the left supraclavicular fossa and mediastinum. Progression-free survival with first-line treatment was 9 months. Because of disease progression, from January 25th to May 30th,2022, the patient underwent the second line treatment of another six cycles of etoposide and cisplatin regimen resulting in a best response classified as PR. However, disease progression was detected through CT scan on June 17th, 2022. In subsequent third-line therapy and later line therapy, Irinotecan followed by Anlotinib (an anti-angiogenesis TKI) were administered, resulting in stable disease. Finally, disease progression occurred on February 18th, 2023 and unfortunately, the patient passed away succumbed to disease progression on April 16th, 2023 with overall survival of more than two years (**Figure 4**).

Overall, this patient exhibited good treatment tolerability. During the six cycles of first-line chemotherapy using toripalimab in combination with etoposide and cisplatin, the patient successfully completed the treatment without experiencing significant toxic effects. His physical condition remained good, with no apparent immunotoxicity or allergic reactions. Monitoring of thyroid function also showed normal results. As the doses of chemotherapy drugs accumulated, during second-line treatment with etoposide and cisplatin, the patient developed mild neurotoxicity and a decrease in white blood cell count post-chemotherapy. However, after receiving symptomatic treatment, the adverse reactions were manageable. In subsequent treatments, the patient did not experience notable adverse reactions such as diarrhea or hypertension, possibly due to the use of pretreatments like atropine. I believe that the patient’s relatively long survival time of two years was attributed to the overall mild toxicity of the treatment and the ability to undergo treatment on time and at the prescribed intensity.

## Collection and review of literatures

Literature search was conducted in the CNKI database up to May 31, 2024 using keywords such as penis, penile metastatic carcinoma, and priapism. No restrictions were placed on the type of literature. The references of included articles were reviewed to minimize the risk of missing relevant studies. In total, 47 documents containing information on 66 cases were retrieved and detailed information were summarized in **Supplementary Material** (**Supplementary Table S1**).

## Discussion

Secondary penile tumor as metastatic disease is rare. Since Eberth reported the first case of rectal cancer penile metastasis in 1870 (2), so far, no more than 600 cases have been reported in the English literature. The common sources of primary tumors are genitourinary system and digestive system. Bladder cancer (28.6%) and prostate cancer (27.9%) are the commonest primary tumors, followed by rectum-sigmoid (12.2%), kidney (6.9%) and lung (6.2%) (3). Except for the urinary system and digestive system, lung is the third common primary tumor origin. Among the different histology types, lung squamous cell carcinoma is the most common pathological type in penile metastasis from lung cancer, which accounts for nearly to 60%. In a retrospective analysis, a total of 40 patients with lung cancer with penile metastasis, 23 patients with lung squamous cell carcinoma, only 2 patients with small cell carcinoma (4). In the Chinese population (**Supplementary Table S1**), the sources of primary tumors were same to that reported in Western countries, the most common were genitourinary system and digestive system tumors, followed by lung cancer. Specifically, among 66 cases of penile metastatic cancer, 18 cases originated from bladder cancer (27.3%), 12cases prostate cancer (18.2%), 3 cases kidney cancer (4.5%), 15 cases colorectal cancer (22.7%), 10 cases lung cancer (21.6%). Among the 10 patients with lung cancer with penile metastasis, 7 cases of squamous cell carcinoma, 1 adenocarcinoma, 1 sarcomatoid carcinoma, 1 epithelioid hemangioendothelioma. This was the first case of small cell lung cancer with penile metastasis reported in the Chinese population.

The mechanisms underlying the spread of tumors to the penis remain unclear. Cherian et al. proposed five main mechanisms: retrograde venous route, retrograde lymphatic route, direct spread, arterial dissemination, and implantation secondary to instrumentation (e.g. prostate resection or urethrotomy) (5, 6). Retrograde venous route is considered the most plausible mechanism for cancer metastasis to the penis due to the communication between the dorsal penile venous system and venous plexuses draining the pelvic viscera facilitating easy dissemination of malignant cells. This explains why bladder cancer, prostate cancer, rectal cancer, and other pelvic tumors are commonly observed as primary tumors with penile metastasis. Among the 66 patients reviewed in this study, primary pelvic tumors accounted for 71.2% (47/66), including 18 cases of bladder cancer, 12 cases of prostate cancer, 15 cases of rectal cancer, 1 case of perineum malignant melanoma and 1 case of testicular cancer. These findings align with reports from non-Chinese populations.

Superficial skin ulceration is the most frequently observed clinical manifestation of primary penile cancer. However, in patients with secondary penile cancer, penile nodule and malignant priapism are the two predominant symptoms, followed by urinary symptoms (including urethral hemorrhage, hematuria, incontinence, irritative, obstructive symptoms) and pain (7). In cases where pelvic bone metastasis is present, bone pain may also manifest. The clinical manifestations of metastatic carcinoma of the penis were similar among Chinese individuals. Among Chinese cases, penile nodule was identified as the most common initial symptom (69.7%, 46/66), followed by malignant priapism (18.2%, 12/66). Penis pain (37.9%, 25/66), dysuria (12.1%, 8/66), and penile swelling (4.5%, 3/66) often coexist with these aforementioned symptoms. The patient in this particular case was also diagnosed with a penile nodule as their first symptom. Although subsequent lung cancer metastasis was discovered and the disease had progressed, this patient did not exhibit any chest-related symptoms. Interestingly, upon reviewing relevant cases, we found similar situation where patients with penile metastasis originating from lung cancer presented with penile lesions without complaining of lung-related symptoms (8). This differs from cases involving penile metastases originating from primary tumors of the urinary or digestive system which penile metastasis occurred after a period following diagnosis and treatment of the primary tumor. Among 10 cases of lung cancer with penile metastasis in Chinese population, 7 cases were diagnosed as lung cancer by complaining of penile lesions.

When presented with penile nodules or malignant priapism, especially in the presence of a history of other cancers, doctors should consider the lesion as metastatic rather than penile ulcer lesions. To further confirm and identify the pathological type, biopsy or fine-needle aspiration should be performed on the lesion. If possible, obtaining pathological data from the primary lesion is encouraged. In this case, we obtained a specimen of the penile lesion through fine needle aspiration and a specimen of the pulmonary lesion through bronchoscopy biopsy. Both specimens suggested small cell carcinoma, and immunohistochemistry analysis showed positive expression of neuroendocrine-related markers such as Syn, CgA, CD56 in both specimens. The specimen from the penis lesion also tested positive for TTF1. These findings support a diagnosis of penile metastasis from small cell lung cancer. Noninvasive examinations such as color Doppler ultrasound, CT scan, MRI scan and positron emission tomography (PET) are effective in evaluating the disease by assessing tumor lesions’ relationship with adjacent structures and searching for primary lesions and distant metastases.

Secondary penile cancer can be managed through various treatment modalities including chemotherapy, targeted therapy, endocrine therapy, surgical intervention, radiotherapy, or a combination of these approaches. The selection of the treatment plan primarily depends on factors such as the patient’s overall health status, histopathological subtype of the tumor, location and size of the primary tumor, extent of metastasis, previous treatments received, and desired treatment goals; however, it is important to note that the prognosis for this condition is generally unfavorable. In order to alleviate or eliminate penile pain associated with secondary penile cancer, options such as penile resection or dorsal penile neurotomy may be considered.

The treatment process of the patient in this case demonstrated good tolerability and positive therapeutic effects. During the first-line treatment phase, the patient responded well to the combination therapy of toripalimab and chemotherapy, without experiencing significant toxicity or immunotoxicity. During the second-line treatment phase, mild neurotoxicity and leukopenia occurred, which were controlled after symptomatic treatment. In subsequent treatments, the use of pretreatment medications prevented the patient from experiencing significant adverse reactions such as diarrhea. Furthermore, the patient’s overall toxicity was not severe, allowing him to receive treatment on time and at the intended intensity, which was also a crucial factor in his long-term survival. This suggests that in clinical treatment, we should tailor treatment plans according to the specific conditions of patients and closely monitor their treatment responses and adverse reactions, adjusting the treatment plan in a timely manner to improve patient outcomes and quality of life.

Most patients diagnosed with secondary penile cancer presented with widespread systemic metastases. Consequently, the main focus of management is palliative care aimed at improving quality of life since long-term survival rates are typically limited to approximately one year following diagnosis from onset of penile metastasis until death occurs (ranging from 0.75 to 27 months in our reviewed cases). Penile metastasis from small cell lung cancer is very rare, another literature summarized 44 cases of penile metastasis from lung cancer, only 2 cases of small cell lung cancer, one of which had an overall survival of more than 12 months, the other had an overall survival of only 3 months (9), and the overall survival of our case was more than 2 years, which is also the highlight of our case and was indicative of good therapeutic efficacy. This positive outcome was attributed to the appropriate treatment regimen and relatively low treatment-related toxicity.

## Conclusion

In summary, this rare disease known as secondary penile cancer often originates from primary tumors located within either genitourinary system or digestive system. The unique characteristic distinguishing it from other forms of penile metastases lies in its simultaneous or even pre-existing diagnosis alongside lung cancer. It is suggested that lung cancer with penile metastasis has high metastasis potential. No specific preferred treatments have been identified based on available evidence; however, systemic treatment for primary cancer is recommended.

## Data Availability

The original contributions presented in the study are included in the article/**Supplementary Material**. Further inquiries can be directed to the corresponding author.

## Supplementary material

Supplementary Figure 1 Flexible bronchoscopy image shows neoplasm in bronchi of left upper lobe with the second carina involved. **(A)** carina. **(B)** left main bronchus. **(C)** the opening of upper and lower of left lobe. **(D)** the left upper lobe.

## References

[1] Sung H Ferlay J Siegel RL Laversanne M Soerjomataram I Jemal A. Global cancer statistics 2020: GLOBOCAN estimates of incidence and mortality worldwide for 36 cancers in 185 countries. CA Cancer J Clin. (2021) 71:209–49. doi: 10.3322/caac.21660 33538338

[2] Eberth C. Krebsmetastasen des Corpus cavernosum penis. Virch Arch. (1870) 51:145–46. doi: 10.1007/bf01879422

[3] Mearini L Colella R Zucchi A Nunzi E Porrozzi C Porena M. A review of penile metastasis. Oncol Rev. (2012) 6:e10. doi: 10.4081/oncol.2012.e10 25992200 PMC4419641

[4] Guo LC Li G Wang XM Zhang M Huang JA Chen YB. Penile metastases from primary lung cancer: Case report and literature review. Med (Baltimore). (2017) 96:e7307. doi: 10.1097/MD.0000000000007307 PMC5500058 28658136

[5] Abeshouse BS Abeshouse GA. Metastatic tumors of the penis: a review of the literature and a report of two cases. J Urol. (1961) 86:99–112. doi: 10.1016/S0022-5347(17)65117-6 13681031

[6] Cherian J Rajan S Thwaini A Elmasry Y Shah T Puri R. Secondary penile tumours revisited. Int Semin Surg Oncol. (2006) 3:33. doi: 10.1186/1477-7800-3-33 17032461 PMC1618838

[7] Chan PT Bégin LR Arnold D Jacobson SA Corcos J Brock GB. Priapism secondary to penile metastasis: a report of two cases and a review of the literature. J Surg Oncol. (1998) 68:51–9. doi: 10.1002/(sici)1096-9098(199805)68:1<51::aid-jso11>3.0.co;2-u 9610664

[8] Zheng FF Zhang ZY Dai YP Liang YY Deng CH Tao Y. Metastasis to the penis in a patient with adenocarcinoma of lung, case report and literature review. Med Oncol. (2009) 26:228–32. doi: 10.1007/s12032-008-9113-8 18975150

[9] Yan W Fu H Liu H Liu Z Qi X Chen T. Acute urinary retention due to corpus cavernosum penile metastasis from lung adenocarcinoma after targeted therapy: a case report and review of the literature. Front Oncol. (2024) 14:1278245. doi: 10.3389/fonc.2024.1278245 38496763 PMC10940509

