# Supplementary figures and images for: A patient with penile metastasis secondary to small cell lung cancer successfully treated with PD-1 inhibitors and chemotherapy: a case report and literature review
Source: Front Oncol. 2025 Feb 25;15:1484365. doi: 10.3389/fonc.2025.1484365 (PMC11893422; doi:10.3389/fonc.2025.1484365)

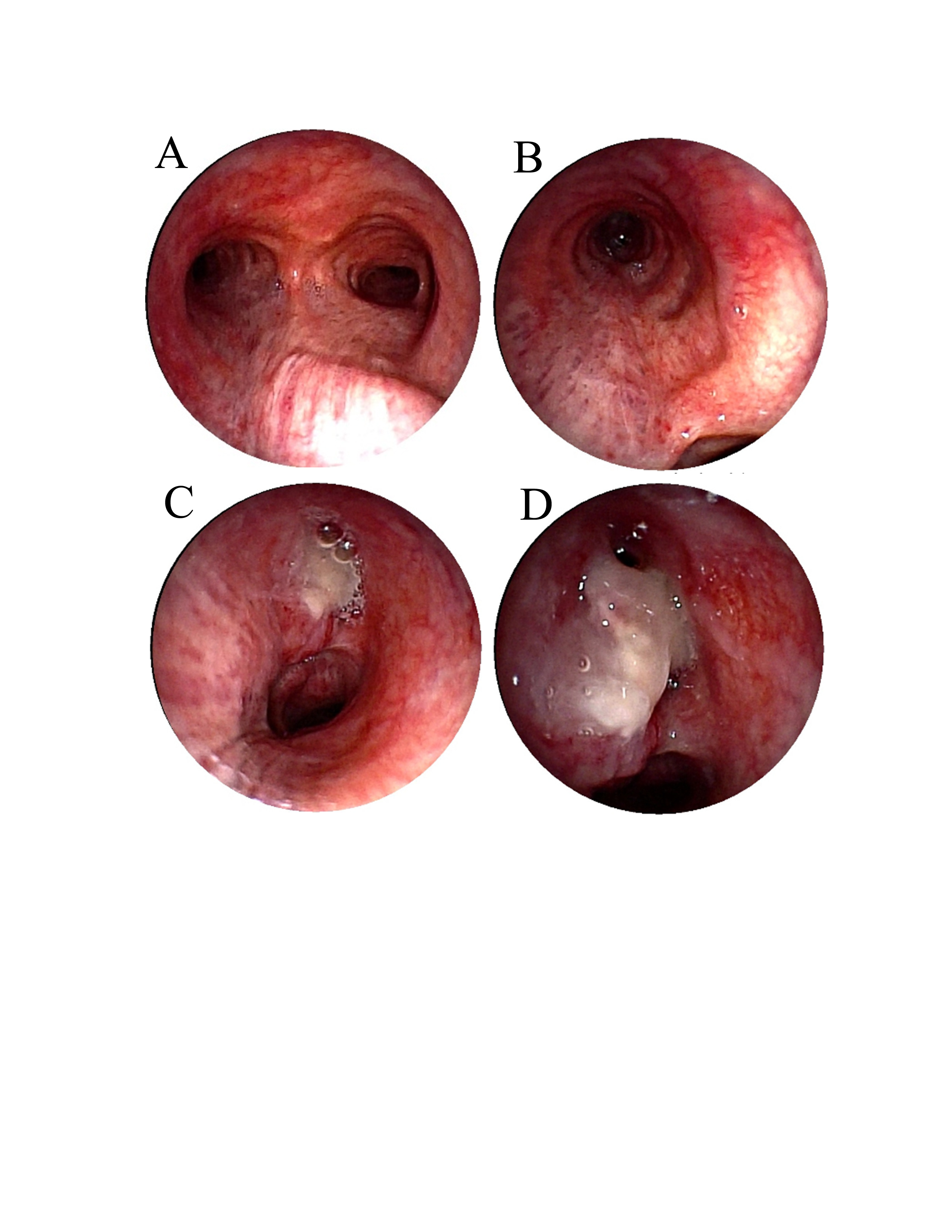

Supplement: Supplementary Figure 1 — Flexible bronchoscopy image shows neoplasm in bronchi of left upper lobe with the second carina involved. (A) carina. (B) left main bronchus. (C) the opening of upper and lower of left lobe. (D) the left upper lobe. [file Image1.jpeg]
