# Supplementary material for: A patient with penile metastasis secondary to small cell lung cancer successfully treated with PD-1 inhibitors and chemotherapy: a case report and literature review
Source: Front Oncol. 2025 Feb 25;15:1484365. doi: 10.3389/fonc.2025.1484365 (PMC11893422; doi:10.3389/fonc.2025.1484365)
Supplement: Supplementary file 2 [file Table1.docx]

**Table S1. Summary of penile metastasis in Chinese population.**

| Number | Authors | Age (years) | Clinical symptoms | Histopathology | Location of primary lesion | Other metastases coexisted | Interval between diagnosis and penile metastasis (months) | Treatments | OS after penile metastasis (months) |
| --- | --- | --- | --- | --- | --- | --- | --- | --- | --- |
| 1 | Zhang et al(1) | 71 | Penis swelling | Muscle-invasive transitional cell  carcinoma of the urinary bladder | Bladder | Two-side inguinal lymph node | 12 | Chemotherapy, radiotherapy and I^125^ particle insert | NR |
| 2 | Lu et al(2) | 73 | Malignant priapism | Transitional cell  carcinoma of the urinary bladder | Bladder | Liver, lung | NR | Shunt of head of penis and cavernous body, Cavernous body of penis urethral cavernous body fenestration shunt, total penis resection | 4 |
| 3 | Li et al(3) | 52 | Penile nodule and pain | Transitional cell  carcinoma of the urinary bladder | Bladder | NR | 4 | None | 3 |
| 4 | Wang et al(4) | 66 | Penile pain | Transitional cell  carcinoma of the urinary bladder | Bladder | - | 36 | Partial Penis Resection | NR |
| 5 | Wang et al(5) | 79 | Malignant priapism, penile pain | Transitional cell  carcinoma of the urinary bladder | Bladder | - | 31 | Chemotherapy * 4 (gemcitabine, cisplatin) | 12 |
| 6 | Chen et al(6) | 51 | Malignant priapism | Transitional cell  carcinoma of the urinary bladder | Bladder | Two-side inguinal lymph node | 132 | Chemotherapy | >12 |
| 7 | Yang et al(7) | 68 | Penile mass, tenderness | Transitional cell  carcinoma of the urinary bladder | Bladder | - | >8 | Total penis resection | 5 |
| 8 | Yang et al(7) | 64 | Penile mass | Transitional cell  carcinoma of the urinary bladder | Bladder | - | >6 | Total penis resection | 4 |
| 9 | Hu et al(8) | 71 | Penile mass | Low-moderately differentiated squamous cell carcinoma | Bladder | - | >12 | Total penis resection, radiotherapy, | >17 |
| 10 | Guo et al(9) | 60 | Penile nodule | Transitional cell  carcinoma of the urinary bladder | Bladder | - | 5 | Total penis resection, Prepubic lymph node dissection, radiotherapy | >16 |
| 11 | Wei et al(10) | 57 | Penile nodule | Transitional cell  carcinoma of the urinary bladder | Bladder | Lymph node | 18 | Total penis resection, pelvic mass resection, radiotherapy | 12 |
| 12 | Xu et al(11) | 71 | Penile mass | Adenocarcinoma | Prostate | Bone | 26 | Bilucaramide, Radionuclide | NR |
| 13 | Zhang et al(12) | 68 | Penile mass, dysuria and pain | Poor differentiated carcinoma | Prostate | Lung | 20 | Radiotherapy, castration treatment, chemotherapy | >18 |
| 14 | Ma et al(13) |  | Penile nodule and ulcer | Murkel cell carcinoma | Prostate | Liver, lymph node | 12 | Chemotherapy (Docetaxel), Estramustine | >12 |
| 15 | Luo et al(14) | 73 | Penile nodule and ulcer | Adenocarcinoma | Prostate | Bone | 0 | Partial Penis Resection | >9 |
| 16 | Wang et al(15) | 69 | Dysuria | Moderate differentiated adenocarcinoma | Prostate | Bone, lung | 1 | Radiotherapy | NR |
| 17 | Li et al(16) | 76 | Penile mass, dysuria | Poor differentiated adenocarcinoma | Prostate | Bone | 0 | Total penis resection, flutamide, Radionuclide | NR |
| 18 | Luo et al(17) | 68 | Malignant priapism | Adenocarcinoma | Prostate | Bone, two-side inguinal lymph node | 0 | Winter shunt, endocrine treatment | 13 |
| 19 | Zhang et al(18) | 34 | Penile nodule, malignant priapism | Papillary renal cell carcinoma | Right kidney | - | 0 | α-IFN | >3 |
| 20 | Fang et al(19) | 49 | Penile nodule, malignant priapism | Clear cell carcinoma | Left kidney | Auxiliary para-arterial lymph nodes | 1.5 | α-IFN | NR |
| 21 | Yuan et al(20) | 69 | Malignant priapism, dysuria, penile nodule | Clear cell carcinoma | Left kidney | - | 1 | Winter shunt, sorafenib | NR |
| 22 | Zhen et al(21) | 65 | Dysuria, penile pain, penile nodule | Adenocarcinoma | Rectum | Lung, liver, retroperitoneal lymph node | 16 | Radiotherapy, chemotherapy | 11 |
| 23 | Zheng et al(22) | 54 | Penile nodule | Adenocarcinoma | Rectum | Liver, retroperitoneal lymph node | 18 | Oxaliplatin | NR |
| 24 | Zhang et al(23) | 53 | Penile nodule and ulcer | Adenocarcinoma | Rectum | Lung, liver | 12 | NR | NR |
| 25 | Ma et al(24) | 74 | Penile nodule | Poor differentiated adenocarcinoma | Rectum | - | 48 | Palliative care | 10 |
| 26 | Ma et al(24) | 55 | Penile pain and malignant priapism, penile nodule | NR | Rectum | Lung, liver | 60 | Palliative care | 6 |
| 27 | Xu et al(25) | 56 | Penile mass | Poor differentiated adenocarcinoma | Rectum | Right testis | 48 | Total testis and penis resection | >12 |
| 28 | Xu et al(25) | 57 | Dysuria | Adenocarcinoma | Rectum | Liver | 36 | Total penis resection | NR |
| 29 | Xu et al(26) | 47 | Penile nodule and pain | Mucinous adenocarcinoma | Rectum | Lung, pleura | 19 | Lumpectomy | 2 |
| 30 | Lai et al(27) | 63 | Penile mass | Moderate differentiated adenocarcinoma | Rectum | Liver | 3 | None | NR |
| 31 | Lu et al(28) | 62 | Penile nodule | Adenocarcinoma | Rectum | - | 0 | None | NR |
| 32 | Luo et al(29) | 54 | Penile nodule, dysuria | Adenocarcinoma | Rectum | Liver | 0 | Chemotherapy | 10 |
| 33 | Mu et al(30) | 46 | Penile nodule and pain | Mucinous adenocarcinoma | Rectum | - | 36 | Chemotherapy, radiotherapy | NR |
| 34 | Wei et al(10) | 51 | Penile nodule | Adenocarcinoma | Rectum | - | 5 | Penile cavernectomy, chemotherapy, radiotherapy | NR |
| 35 | Wei et al(10) | 75 | Penile nodule | Squamous cell carcinoma | Lung | - | 0 | Chemotherapy | 6 |
| 36 | Yu et al(31) | 81 | Penile pain and swelling | Squamous cell carcinoma | Lung | - | 0 | Radiotherapy | 3 |
| 37 | Cheng et al(32) | 76 | Malignant priapism, dysuria | Squamous cell carcinoma | Lung | - | 0 | Winter shunt | 0.75 |
| 38 | Liu et al(33) | 55 | Penile mass | Squamous cell carcinoma | Lung | - | 5 | None | 6 |
| 39 | Li et al(34) | 49 | Penile mass | Squamous cell carcinoma | Lung | Right adrenal gland | 6 | Gefitinib | >14 |
| 40 | Xiang et al(35) | 68 | Malignant priapism | Squamous cell carcinoma | Lung | - | 0 | None | 12 |
| 41 | Xiang et al(35) | 51 | Penile ulcer and pain | Adenocarcinoma | Lung | Inguinal lymph node, bone | 0 | Penis resection, chemotherapy (Gemcitabine, cisplatin) | 3 |
| 42 | Xiang et al(35) | 46 | Penile pain | Sarcomatoid carcinoma | Lung | - | 0 | None | 12 |
| 43 | Shan et al(36) | 67 | Penile nodule, urine retention | Squamous cell carcinoma | Lung | - | 36 | Total penis resection, chemotherapy, radiotherapy | 6 |
| 44 | Shan et al(36) | 62 | Penile nodule and pain | Epithelioidhemangioendothelioma | Lung | - | 7 | Total penis resection | 2 |
| 45 | Shan et al(36) | 88 | Penile nodule | High grade urothelial cell carcinoma | Bladder | - | 11 | Total penis resection, irrigation of bladder | 5 |
| 46 | Shan et al(36) | 68 | Penile nodule and pain | High grade urothelial cell carcinoma | Bladder | - | 17 | Total penis resection, irrigation of bladder | >18 |
| 47 | Shan et al(36) | 44 | Malignant priapism | Hemangioma | Liver | Lung, bone, retroperitoneal lymph node | 16 | Total penis resection | 3 |
| 48 | Shan et al(36) | 66 | Penile nodule and pain | Adenocarcinoma | Prostate | Liver, bone | NR | Endocrine therapy | 8 |
| 49 | Li et al(37) | 25 | Malignant priapism | Germ cell tumor | Testis | Lung, mediastinal lymph node | 0 | Winter shunt | >7 |
| 50 | Zhou et al(38) | 55 | Penile ulcer | Adenocarcinoma | Cardia | - | 0 | Partial penis resection | NR |
| 51 | Zheng et al(39) | 42 | Penile mass | Hepatic celluler cancer | Liver | - | 0 | None | 7 |
| 52 | Cui et al(40) | 35 | Penile nodule | T-lymphocyte lymphoma | Nasopharynx | - | 29 | Partial penis resection | NR |
| 53 | Zhou et al(41) | 39 | Penile mass | enteric adenocarcinoma | Bladder | Lugn、bone | 4 | Chemotherapy(carboplatin, Paclitaxel)，immunotherapy | NR |
| 54 | Du et al(42) | 72 | Dysuresia | Adenocarcinoma | rectum | - | NR | NR | NR |
| 55 | Wang et al(43) | 63 | penile nodule | Squamous carcinoma | esophagus | lymph node, thigh | 10 | None | 2 |
| 56 | Zhang et al(44) | 63 | Penile nodule and pain | Basal cell carcinoma | prostate | - | 6 | Partial penectomy | NR |
| 57 | Yan et al(45) | 64 | Penile mass | malignant melanoma | perineum | - | 30 | Partial penectomy | NR |
| 58 | Yan et al(45) | 78 | Penile mass and pain | Urothelial carcinoma | Bladder | - | 58 | Total penis resection | 9 |
| 59 | Yan et al(45) | 67 | Penile pain | adenocarcinoma | colon | Lung、liver | 3 | Total penis resection，Chemotherapy | 6 |
| 60 | Yan et al(45) | 76 | Penile enlargement | Peripheral NK/T cells lymphoma | lymph gland | - | 0 | Chemotherapy | 27 |
| 61 | Yan et al(45) | 82 | Penile pain | Urothelial carcinoma | Bladder | - | 8 | radical penectomy | 7 |
| 62 | Wang et al(46) | 69 | Penile pain | Urothelial carcinoma | Bladder | Lymph node | 72 | Chemotherapy, radiotherapy | 22 |
| 63 | Wang et al(46) | 66 | Penile pain | Urothelial carcinoma | Bladder | Lymph node | 10 | Total penis resection，Chemotherapy，immunotherapy | NR |
| 64 | Qian et al(47) | 81 | Penile nodule | Adenocarcinoma | Prostate | bone | 32 | Endocrine therapy, radiotherapy | 11 |
| 65 | Qian et al(47) | 78 | Penile mass and pain | Adenocarcinoma | Prostate | Bone | 23 | Total penis resection，Endocrine therapy, radiotherapy | 10 |
| 66 | Qian et al(47) | 79 | Penile mass and pain | Adenocarcinoma | Prostate | Bone | 0 | Endocrine therapy | 9 |

OS=overall survival; NR: not recorded; α-IFN: alpha interferon.

**References:**

1. Zhang JG, Wu Y. Yi li pangguang ai genzhi qiechu shuhou yinjing zhuanyi de fangshe zhiliao. [Radiation therapy for penile metastasis after radical resection of bladder cancer.](in Chinese)Clinical Research. (2014) 22(10):162-162.163.

2. Lu C, Zhang S, Gu X, Pan W, Tong F. Priapism due to metastasis of bladder cancer to penis(a case report and literature review). Chinese Journal of Andrology (2006) 08:34-5,8.

3. Li T, Zhang F.Pangguang yixing xibao ai yinjing haimianti zhuanyi yi li.[ A case report of corpus cavernosum metastasis from bladder transitional cell carcinoma.] Chinese Journal of Andrology (1996) 02:19.

4. Wang Q, Lu J, Huang S. Pangguang yixing xibao ai yinjing zhuanyi 2 li baogao. [Penile metastasis from bladder transitional cell carcinoma: two cases report.] Chinese Journal of Urology (2004) 25(5):321-321.

5. Wang Z, Zhu Y, Xiao J, Guo Y, Tian Y, Lin J. Gemcitabine combined with cisplatin in the treatment of penile metastasis of bladder cancer: a case report. Chin J Urol (2017) 25(1):80-80.

6. Chen G, Zhu X, Gao W, Cai S, Liang Z, Shen Y. Diagnosis and Treatment of Priapism Due to Neoplasma of Penis. National Journal of Andrology (2006) 12(2):162-163

7. Yang C, Wang Y, Bi J, Kong C. Diagnosis and Treatment of Penis Metastatic Carcinoma of Bladder Cancer: Two Cases Report. Journal of China Medical University (2014) 43(10):952-3.

8. Hu Z, Xie Q. Diagnosis and Treatment of Penis Metastatic Squamous Carcinoma of Bladder: One Case Report. Chinese General Practice (2012) 15(09): 1032-1033.

9. Guo Q, Liang C, Liu M, Ye Y, Wang K, Li H. Penile metastasis secondary to transitional cell carcinoma of the urinary bladder (a case report and review of the literature). Chin J Urol (2006) 27(11): 772-775.

10. Wei D, Wan B, Zhang L, Liu M, Zou E. yinjing zhuanyi ai 3 li baogao:fuxi wenxian tantao gai bing d e zhenduan, zhiliao he yuhou.[3 cases Report of metastatic carcinoma of the penis: literature review to explore the diagnosis, treatment and prognosis of the disease.] Tumor (2004) 24(5): 516.

11. Xu X, Huang Q, Jiang S. Qianliexian ai ban yinjing tou zhuanyi 1 li baogao bing wenxian fuxi. [Prostate Cancer with Glans Penis Metastasis: Case Report and Literature Review.] J Clin Urology (China) (2016) 31(04): 379-381.

12. Zhang Z, Bai G, Yu W, He Q, Song Y, Jin J. Comprehensive treatment of prostate cancer with penis metastasis: a case report and literature review. Chin J Clinicians (Electronic Edition) (2020) 14(3):217-220.

13. Ma C, Wang H. A case of penile metastasis after electroresection of prostate cancer. J Clin Surg (2008) 16(11):777-777.

14. Luo H, Ding R. Rare metastatic of prostate cancer: report of 4 cases. J Modern Urol (2005) 10(5):290-291.

15. Wang D, Jiang Qin, Chen S, Jiang Z. Qianliexian ai yinjing zhuanyi yili baodao bing wenxian fuxi. [Penile metastasis of prostate cancer: a case report and literature review]. J Bas Clin Oncol (2024) 37(1):1-6.

16. Li R, Guo X, Zhang B. Qianliexian ai yinjing zhuanyi yili baogao.[Penile metastasis of prostate cancer: a case report] Chin J Urol (2006) 27(8):543.

17. Luo W, Wang M, Gou X, He W, Zhang L. Qianliexian ai zhuanyi zhi yinjing yichang boqi 1 li.[ Priapism caused by metastatic prostate cancer: a case report and literature review.] Chin J Andrology (2011) 25(9):64-65.

18. Zhang B, Cai W, Sun L, Wang X, Hong B. Nangxing shen rutou zhuang xibao ai yinjing zhuanyi yi li baogao. [Penile metastasis of cystic renal papillary cell carcinoma: a case report] Chin J Urol (2006) 27(3):177.

19. Fang K, Wang J, Zuo Y, Mai X, Yang Y. Shenai shuhou yinjing zhuanyi 1 li.[ Postoperative penile metastasis of renal carcinoma: a case report] J Clin Urol (2002) 17(8):404.

20. Yuan J, Liu S, Yu Q, Wang Y, Wang Y, An R. Shenai yinjing zhuanyi zhi yinjing yichang boqi 1 li baogao bing wenxian fuxi.[ Priapism caused by renal carcinoma metastasis: a case report and literature review] J Chin Med Uni (2013) 42(1): 91-92．

21. Zhen Z, Shen C, Sun W. Wanqi jiezhi ai yinjing zhuanyi 1 li baodao. [Penile metastasis of advanced rectal cancer: a case report.] Journal of basic and clinical oncology (2012) 25(1):71-72.

22. Zheng J, Qu F, Bai T, Nie J, Zhang Y. Penile metastasis from rectal adenocarcinoma diagnosed by fine-needle aspiration: a case report and literature review. Modern Oncol (2013) 21(1):110-111.

23. Zhang J, Wang Y, Ma Q, Li Y, Zhou H. Yinjing zhuanyi xing zhichang xian ai 1 li.[ Penis metastasis of rectum adenocarcinoma: a case report] CHINA MEDICAL HERALD (2011) 8(30):146-146

24. Ma H, Hao L, Dong Z, Cao N, Ma C. Yinjing zhuanyi ai 2 li baogao.[ Metastatic carcinoma of penis: report of 2 cases.] Medicine and Pharmacy of Yunnan (2002) 23(4):348-349

25. Xu H, Meng T. zhichangai shuhou yinjing zhuanyi 2 li. [Penile metastasis occurred in 2 cases after rectal cancer surgery.] Chinese Journal of Coloproctology (2005) 25(12):27-27

26. Xu T, Chen Y. Zhichang ai yinjing pixia zhuanyi yi li. [A case of rectal cancer with subcutaneous metastasis of penis.] Journal of Practical Oncology (1988) 04 :238.

27. Lai Z, Yang Z, Yu Z. Zhichangai yinjing zhuanyi 1 li. [One case of penile metastasis of rectal cancer]. Chinese Journal of Clinical Oncology and Rehabilitation (1997) 03:78.

28. Lu K, Niu B, Du Y. Zhichangai yinjing zhuanyi 1 li. [One case of penile metastasis of rectal cancer] Chin J Clin Oncol (1998) 03:55.

29. Luo N, Zhang Z, Ma Y. Penile metastasis from rectal carcinoma: A case report and literature review. Natl J of Androl (2014) 20 (4):359-362.

30. Mu K, Liao W, Chen Y, Zhang L. Penile Corpus Cavernosum Metastasis Secondary to Rectal Adenocarcinoma Undergone Miles’ Operation (A Case Report and Literature Review). Chin J Bases Clin General Surg (2011) 18(7):759-761.

31. Yu L, Li H, Zhang S. Feiai shoufa yinjing haimianti zhuanyi ai yi li.[A Case of Primary Lung Cancer with Penile Corpus Cavernosum Metastasis.] Chin J Clinicians (Electronic Edition) (2011) 5(19):5836-5837.

32. Cheng L, Yang W, Cai H, Shi C, Zeng F. Malignant priapism due to lung cancer metastasizing to penis：a case report and literatures review. Chinese Journal of Andrology (2020) 34(2):69-71.

33. Liu S, Sun W, Jiang L. Feilingai yinjing zhuanyi 1 li baogao ji wenxian fuxi. [A Case Report of Penile Metastasis from Lung Squamous Cell Carcinoma and Literature Review.] Natl J of Androl (2013) 19 (3): 286-287.

34. Li R, Wang L, Liu X, Wang L, Qin S. Gefitinib zhiliao feilingai yinjing he shenshangxian zhuanyi 1 li.[A Case Report of Gefitinib Treatment for Lung Squamous Cell Carcinoma with Penile and Adrenal Metastases.] Chinese Clinical Oncology (2006) 11(8):639-639

35. Xiang S, Zhou J, Gan S, Li J, Peng H, Wang S. Clinical features and treatment of penile metastasis secondary to lung malignancies with the initial symptoms of penis. J Clin Urology (China) (2013) 28(12):938-941.

36. Shan Y, Xu M, Wang Z, Zhang K. Penile metastatic carcinoma: a report of 6 cases and literature review. Chin J Clin Medi (2014) 21(6):686-688.

37. Li F, Wang L, Cai X, Chen , Wang T, Ding C. Gaowan zhongliu zhuanyi zhi yinjing yichang boji 1 li baogao.[A Case Report of Priapism Caused by Testicular Tumor Metastasis to the Penis.] Clin J Andrology (2010) 24(2):60.

38. Zhou W, Zhu G. Benmen xianai yinjing guitou bu zhuanyi 1 li. [A Case of Cardia Adenocarcinoma with Metastasis to the Glans Penis.] Chin J Thorac Cardiovasc Surg (2000) 16(5):301.

39. Zheng G, Zou H. Yinjing zhuanyi wei shoufa de yuanfaxing gan ai yi li. [A Case of Primary Liver Cancer with Initial Metastasis to the Penis.] Chin J Radiat Oncol (2003) 12(3):216.

40. Cui H, Liu R. Biyan bu T-Cell Lymphoma yinjing zhuanyi yi li.[A Case of Nasopharyngeal T-Cell Lymphoma with Metastasis to the Penis.] Clinical Misdiagnosis & Mistherapy (1999) 05:397

41. Zhou M, Jiang F, He D, Chen X, Wang R, Liang H, et al. Jing xin fuzhu zhiliao ji genzhi shu hou fei zang he yinjing zhuanyi de pangguang chang xing xian ai bingli taolun. [A case of bladder intestinal adenocarcinoma with lung and penile metastasis after neoadjuvant therapy and radical surgery] J Mod Urol (2022) 27(07):589-92.

42. Du F, Lv Y, Yuan H, Yu Y, Wang T. Zhichang ai zhuanyi yinjing niaodao haimian ti chaosheng zao ying biaoxian 1 li baodao. [Report of one case: Ultrasonic imaging manifestations of rectal cancer metastasis to the penile urethral corpus spongiosum] J Contemp Urol Reprod Oncol (2022) 14(03):178-9.

43. Wang X, Wang N. A case report of penile metastasis of esophageal cance. Journal of Modern Oncology (2021) 29(23):4220-1.

44. Zhang H, Cai Y. Yinjing zhuanyi xing qianliexian jidi xibao ai 1 li.[ One case of penile metastatic of prostate basal cell carcinoma.] J Clin Urology（China）(2020) 35(02):167-8.

45. Yan R, Liang L, Fu X, Bao Y, Liu B, Wang L, et al. Metastatic tumor of penis: 5 cases report and literatures review. Chinese Journal of Andrology (2021) 35(06):79-82.

46. Wang G, Zhang Z, Yu W, Chen C, Song Y, Hao H. Penile metastasis from bladder cancer: a report of 2 cases and review of the literature. Chinese Journal of Clinicians (Electronic Edition) (2021) 15(3):238-240. DOI: 10.3877/cma.j.issn.1674-0785.2021.03.019.

47. Qian Y, Chen Z, Huang C, Lai F, Yang Z, Cai Y, et al. Qianliexian ai yinjing zhuan yi san li baodao bing wenxian fuxi. [Report of Three Cases of Prostate Cancer with Penile Metastasis and Literature Review]. Journal of Practical Oncology (2021) 36(05):448-50.
